# A recent survey on instance-dependent positive and unlabeled learning

**DOI:** 10.1016/j.fmre.2022.09.019

**Published:** 2022-10-12

**Authors:** Chen Gong, Muhammad Imran Zulfiqar, Chuang Zhang, Shahid Mahmood, Jian Yang

**Affiliations:** a School of Computer Science and Engineering, Nanjing University of Science and Technology, Nanjing 210094, China; b PCA Lab, the Key Laboratory of Intelligent Perception and Systems for High-Dimensional Information, Ministry of Education, Nanjing 210094, China; c Department of Computer Science and Information Technology, University of Jhang, Jhang 35200, Pakistan; d Higher Education Department, Punjab, Faisalabad 38000, Pakistan

**Keywords:** Instance-dependent positive and unlabeled learning, Weakly supervised learning, Label noise learning, Cost-sensitive learning, Labeling assumption, Scoring function

## Abstract

Training with confident positive-labeled instances has received a lot of attention in Positive and Unlabeled (PU) learning tasks, and this is formally termed “Instance-Dependent PU learning”. In instance-dependent PU learning, whether a positive instance is labeled depends on its labeling confidence. In other words, it is assumed that not all positive instances have the same probability to be included by the positive set. Instead, the instances that are far from the potential decision boundary are with larger probability to be labeled than those that are close to the decision boundary. This setting has practical importance in many real-world applications such as medical diagnosis, outlier detection, object detection, etc. In this survey, we first present the preliminary knowledge of PU learning, and then review the representative instance-dependent PU learning settings and methods. After that, we thoroughly compare them with typical PU learning methods on various benchmark datasets and analyze their performances. Finally, we discuss the potential directions for future research.

## 1 Introduction

In binary classification, a conventional supervised model is trained on a set of positive data and negative data. In contrast, Positive and Unlabeled learning (PU learning) works on the training set which only contains some labeled positive instances and many unlabeled instances. Here unlabeled instances can be positive or negative ones, but their real labels are unknown to the learning algorithm [1], [2]. Therefore, the main difference between PU leaning and traditional supervised learning is that a PU learning algorithm is not accessible to the explicitly labeled negative instances.

PU learning is quite effective when the negative training instances are missing or extremely diverse. Recently, PU learning has attracted intensive attention, because PU data naturally appear in many important applications. For example:

(1) **Medical diagnosis:** The medical record of a certain patient only contains the diagnosed diseases of the patient in the history, but does not include diseases that the patient does not suffer from. If a patient is not diagnosed with a specific disease in the medical record, it does not mean that the patient has no such disease [3].

(2) **Fake comment detection:** The fake comment detection system of a shopping website can only identify certain definite fake comments, (*a.k.a.* positive instances), but cannot return valid or real comments [4], [5]. Consequently, there is only a small portion of positive data available, and the rest of the unlabeled remarks can be real or fake, so PU learning can be used to construct more accurate detector to distinguish fake comments from the real ones.

(3) **Remote sensing:** In remote sensing, we may only focus on identifying a particular type of land (*e.g.*, vegetation for monitoring the forest expansion) from a hyperspectral image [6]. In this case, the negative class representing “non-forest areas” are diverse, so it is difficult to adequately collect various representative non-forest areas.

(4) **Multi-label learning:** In multi-label learning, it is often the case that the provided labels are incomplete and the absence of a label does not imply that this label is not proper for the example. Therefore, PU learning can be employed to discover the hidden correct labels based on the known labels [7], [8].

From the above examples, we see that PU learning is very important in solving many real-world problems. In fact, most of the conventional PU learning approaches assume that the positive-labeled data are uniformly picked up from the positive distribution in a random way [9], [10]. However, in many practical applications of PU learning nowadays, the positive data are not uniformly generated any more. Instead, they are selected in a biased way [11], [12], [13]. For example, in disease diagnosis, the doctors are more likely to annotate the cases that are definitely illness or healthy. Therefore, instance-dependent PU learning has gained much attention which assumes that whether a positive example will be observed depends on its feature. One simple case is that a data point that is far away from the potential decision boundary has a larger probability to be annotated.

Inspired by these important applications, researchers are very interested in analyzing instance-dependent PU learning settings and have devised a variety of techniques to solve this problem. Without going too deep, our survey firstly introduces traditional PU learning (Section 2), and then provides a comprehensive review on instance-dependent PU learning regarding labeling mechanism (Section 3), typical algorithms (Section 4), relationship with other fields of machine learning (Section 5), and empirical comparisons on some benchmark datasets (Section 6).

In fact, there are several existing literature surveys on PU learning, such as [14], [15], [16], [17]. However, they only review the conventional instance-independent PU learning without touching the recent advances in more realistic instance-dependent PU learning. Therefore, we want to use this survey to summarize the recent progresses on instance-dependent PU learning and draw more researchers attention on this useful and interesting topic.

Some major notations that will be later used are displayed in Table 1.

**Table 1:** **Important symbols used in this survey**.

Symbols	Definition
x	The feature vector of an instance
y	True label of an example
$\bar{y}$	Observed label of an example
s	Indicator variable for labeled instance, where
	s=1 means it is labeled, and 0 otherwise.
c=P(s=1|y=+1)	Label frequency of positive data
P(x|y=+1)	Class conditional distribution
α=P(y=+1)	Class prior of positive data

## 2 A brief review on PU learning

Instance-dependent PU learning is a particular setting of PU learning. Therefore, before formally introducing instance-dependent PU learning, we shall briefly review the setting of traditional PU learning by discussing the generation process of PU training data and the existing methods for exploiting unlabeled data.

### 2.1 Training set generation

Most of the methods developed for PU learning follow two well-known scenarios to generate positive data and unlabeled data, namely, case-control scenario [18] and censoring scenario [17]. The algorithms developed under these two scenarios are displayed in Table 2.

**Table 2:** **Summary of algorithms under different scenarios**.

case-control scenario	[1], [18], [19], [20], [21], [2], [22], [23], [24], [25]
Censoring scenario	[11], [17], [26], [27], [28], [29], [30], [31], [32]

**Case-control scenario:** This scenario is based on a two-sample configuration. In this scenario, the positive instances in the positive set $S_{P}$ and the unlabeled instances in the unlabeled set $S_{U}$ are independently drawn from the class conditional distribution P(x|y=+1) and the marginal distribution P(x), respectively, namely [11], [22], [29],

(1) $$\left\{ \begin{array}{c} S_{P}={\left\{x_{i}\right\}}_{i=1}^{k}\overset{i.i.d.}{∼}P\left(x|y=+1\right)\\ S_{U}={\left\{x_{i}\right\}}_{i=k+1}^{n}\overset{i.i.d.}{∼}P\left(x\right) \end{array}\right.$$

where k is the size of set $S_{P}$ and the size of set $S_{U}$ is n−k.

**Censoring scenario:** This scenario is based on a one-sample configuration, so it is also known as single-training-set scenario. In this scenario, the positive instances and unlabeled instances are drawn from the same set $S={\left\{x_{i}\right\}}_{i=1}^{n}$, where n represents the size of S. A fraction α from the positive instances (instances with actual hidden label +1) are selected to construct the positive set, while the other fraction 1−α as well as all negative instances (instances with actual hidden label −1) are used to construct the unlabeled set. In other words, if the actual hidden label of instance x is +1, it will be labeled with the probability of α. If the actual hidden label of instance x is −1, such instance will never disclose its label, and these instances will belong to the set $S_{U}$ with probability 1.

From the descriptions above, we see that both case-control and censoring scenarios can generate a set of labeled positive examples and unlabeled examples. However, the underlying distributions for generating positive examples are different. Specifically, case-control scenario adopts a two-sample setting and the positive data are generated from the conditional probability P(x|y=+1). In contrast, censoring scenario follows a one-sample setting and assumes that both positive and unlabeled data are generated from the marginal distribution P(x), where positive data are disclosed with a probability of α [11], [29].

### 2.2 Methods of exploiting unlabeled data in traditional PU learning

The commencing study on PU learning reveals the truth that even without the access to explicitly labeled negative data, the unlabeled data has a huge impact on the accurate training of a binary classifier [33]. There are three well-known strategies for exploiting the unlabeled data in PU learning methods, namely, two-step strategy, cost-sensitive strategy, and one-sided label noise conversion strategy.

Just as the name implies, the two-step strategy consists of two steps. The first step is the identification of reliable negative instances from the unlabeled set [34]. The reliable negatives can be defined as the instances that are completely different from the labeled positive ones [35], [36]. Regarding these instances, we are pretty sure that they are not positive instances. In the second step, suitable classifier is applied to the dataset with positive instances and the detected reliable negative instances to perform traditional supervised learning [1], [32], [37]. Some representative works of two-step technique are [38], [39], [40]. The prime goal of this technique is to correctly identify reliable negative instances. The drawback is also obvious, namely, incorrect recognition of reliable negative instances would lead to a substantial decrease of the algorithm performance.

In cost-sensitive PU learning technique, the training instances are properly reweighted. As a result, the observed biased data distribution carried by the PU training set can be calibrated by reweighting the training instances, so that the actual data distribution can be estimated. Here class prior (*e.g.*, α=P(y=+1)) plays an important role. Unfortunately, the prior is usually unknown in advance and should be pre-estimated [19], [41], [42]. The representative approaches include Weighted Logistic Regression [32], [43], Cost-sensitive positive and unlabeled learning [23] and Weighted Support Vector Machine [17], [44] which adjust the weights of data by applying different pre-defined rules. However, the adjustment of weighting parameters is tricky, which may lead to poor performance of the model [30]. The most recent works focus on designing unbiased risk estimator [19], [20], [21]. Such methods are able to avoid the defects associated with adjustment of the weighting parameters and also achieve the improved performance.

The third category treats unlabeled instances as negative instances and then transform the PU learning problem into a label noise learning problem [45], [46], [47]. That is to say, all the labeled positive instances are positive and are truly labeled. However, all unlabeled instances are considered as negative, so the positive instances in the unlabeled set are mistakenly labeled as negative. The noise lies in only observed negative class. Therefore, we say that the noise is one-sided [48]. For example, Up to now, various techniques are developed to eliminate one-sided label noise. For example, Gong et al. and Shi et al. [29], [30] treat all unlabeled instances as noisy negatives and then find an unbiased risk estimator via loss decomposition and centroid estimation.

## 3 Labeling assumptions of instance-dependent PU learning

Instance-dependent PU learning is a particular setting of PU learning which can be enabled by making certain compulsory assumptions of training set generation process and adopting some specific labeling techniques. In this section, we will review in detail the assumptions about the labeling mechanism for instance-dependent PU learning.

### 3.1 Selected completely at random

Selected Completely at Random (SCAR) labeling mechanism considers a set of labeled instances as a consistent subset of the positive set [17], which means that the instances are selected randomly from the positive distribution, regardless of their attributes. The probability of choosing a positive instance e(x) is a constant which is equivalent to the label frequency c, as shown below:

(2) e(x)=P(s=1|x,y=+1)=P(s=1|y=+1)=c

According to SCAR mechanism, the probability of an instance to be labeled is directly proportional to the probability that the instance is positive, namely,

(3) P(s=1|x)=cP(y=+1|x)

Above relationship permits the employment of non-traditional classifiers [49] in instance-dependent PU learning. Non-traditional classifiers can be learned by treating all unlabeled instances as negative with label noise. These classifiers can predict the probability of an instance to be labeled (e.g.,P(s=1|x)). Non-traditional classifiers have the following worth-mentioning features:

• Non-traditional classifiers preserve the property of ranking order among the instances [17], namely,
  $P\left(y=+1|x_{1}\right)>P\left(y=+1|x_{2}\right)\Leftrightarrow P\left(s=1|x_{1}\right)>P\left(s=1|x_{2}\right)$
• If the label frequency c is known, the probabilistic non-traditional classifier can be converted to the traditional classifier by dividing it with label frequency c, namely $P\left(y=+1|x\right)=\frac{P(s=1|x)}{c}$

SCAR mechanism is introduced as an analogy with the Missing Completely at Random (MCAR) hypothesis, which is a common method used when dealing with missing data [50], [51]. In spite of several similarities between both assumptions, there is a significant difference between them. In MCAR hypothesis, the missing variable is not dependent on the value of the variable. Differently, in SCAR mechanism, the missing variable depends on the value of the missing variables, because all the negative instances are missing in the case of instance-dependent PU learning [15]. Kato et al. [24] implemented SCAR assumption in instance-dependent PU learning by developing an average technique for incorporating a distribution over class prior instead of calculating the exact value of the class prior.

### 3.2 Selected at random

Selected at Random (SAR) is a well-known labeling mechanism which assumes that the positive instances are selected randomly from positive distribution and the probability of picking an instance depends on the value of its attributes [52]. SAR labeling mechanism is based on the reality that several real-world applications are affected by the bias. For example, whether a patient having a certain disease visits a doctor depends on his/her financial status and on the severity of his/her disease symptoms. The bias is fully dependent upon the characteristics of the instances [24]. In SAR mechanism, a notion called “propensity score” e(x) is usually employed, which is mathematically defined as e(x)=P(s=1|x,y=+1).

## 4 Algorithms for instance-dependent PU learning

Up to now, there are mainly two well-known types of the algorithms which aim to handle instance-dependent PU learning, namely *Scoring Function Algorithms* and *Bayesian Optimal Relabeling*. Some major algorithms belonging to these two types are summarized in Table 3 and they will be detailed in the following.

**Table 3:** **Taxonomy of existing instance-dependent PU learning methods**.

Scoring function algorithms	[11], [24], [25], [53]
Bayesian optimal relabeling	[27]

### 4.1 Scoring function algorithms

Scoring function is a common tool used in instance-dependent PU learning. Traditional PU learning case can be converted to instance-dependent PU learning by inserting a scoring function. The following are well-known algorithms for instance-dependent PU learning using scoring functions as standard tools.

**PU learning with a Selection Bias (PUSB):** It is quite difficult to learn the Bayesian optimal classifier in the presence of selection bias from the traditional PU learning methods. To tackle this issue, Kato et al. [24] devised a novel algorithm, known as PUSB algorithm. PUSB algorithm learns a scoring function that retains the order caused by the class posterior under mild assumptions, and can be used as a classifier by associating a suitable threshold with it. However, it is impossible to calculate the class posterior in the presence of selection bias, even if the class prior α is known.

Therefore, Kato et al. [24] introduces a new concept of partial identification in their PUSB algorithm in instance-dependent PU learning. According to partial identification, it is better to extract certain valuable information of class posterior P(y=+1|x) instead of calculating the class prior to learn the classifier. Partial identification can be represented by the following equation:

(4) $$r\left(x\right)=\frac{P(x|y=+1,s=1)}{P(x)}$$

where r(x) is the density ratio.

If we can calculate r(·), we can extract the total order of the set caused by P(y∣·), even if we are unable to estimate P(y∣·). For two instances $x_{i}\neq x_{j}$, the characteristic of preserving the order of the scoring function of instances is shown as:

(5) $$P\left(y=+1|x_{i}\right)\leq P\left(y=+1|x_{j}\right)\Leftrightarrow r\left(x_{i}\right)\leq r\left(x_{j}\right)$$

Kato et al. [24] recommended the estimation of r and used it as a scoring function to capture the total order caused by P(y=+1|x). After obtaining the observed value of scoring function $\hat{r}$, a threshold θ∈R was carefully chosen, leading to the final classifier h(x)=sign(r(x)−θ). They also proposed a method to select θ based on data.

At the end, they modified the pseudo classification risk used in traditional PU learning [19], [20] as:

(6) $$R_{PU}^{bias}\left(f,l\right)=\alpha E_{P}^{bias}\left[l\left(f\left(x\right),+1\right)\right]-\alpha E_{P}^{bias}\left[l\left(f\left(x\right),-1\right)\right]-E_{u}\left[l\left(f\left(x\right),-1\right)\right]$$

where $R_{PU}^{bias}\left(f,l\right)$ is the pseudo classification risk used in instance-dependent PU learning, α is the class prior, $E_{p}^{bias}$ is the expectation over p(x|y=+1,s=1), $E_{u}$ is the expectation over P(x), ℓ(·) is a loss function, and f is a decision function in traditional PU learning.

**SAR-PU:** Selected at Random Positive-Unlabeled (SAR-PU) is a common instance-dependent PU learning algorithm designed by Bekker et al. [53]. The propensity score is also used as a scoring function by Gong et al. [11] in their recent study on instance-dependent PU learning. Bekker et al. [53] used SAR labeling mechanism in this algorithm. According to SAR labeling mechanism, the probability of existence of all positive instances is not the same. The probability of a positive instance being labeled depends on its attributes. To enable instance-dependent PU learning, they used a scoring function known as propensity score, of which the concept is taken from a causal inference survey [54]. Propensity score is denoted by e(x) and can be mathematically represented as:

(7) e(x)=P(s=1|y=+1,x)

The propensity score is limited to the positive class only, which is the biggest difference from the causal inference. Unlike the causal inference, the negative instances are not weighted with propensity score in instance-dependent PU learning, because the probability of labeling of negative instances is zero in instance-dependent PU learning. For each labeled instance with a propensity score e(x), it is expected that there would be $\frac{1}{e_{i}}$ positive instances, of which $\frac{1}{e_{i}-1}$ are not selected for labeling. This approach adopts count to calculate the accurate number of instances along-with their relevant propensity score from the observed positive instances.

Propensity scores can only be learnt from PU data by making certain assumptions: for example, if the propensity score of a random instance is small, it is impossible to know whether an instance is labeled or not. Therefore, the propensity score needs to rely on fewer attributes than the finally output classifier [55]. One of the simplest methods to learn propensity score is to consider that the propensity function depends on the propensity attributes, which are the subset of attributes, namely,

(8) $$\left\{ \begin{array}{c} P\left(s=1|y=+1,x\right)=P\left(s=1|y=+1,x_{e}\right)\\ e\left(x\right)=e\left(x_{e}\right), \end{array}\right.$$

where $x_{e}$ is the propensity attribute.

The propensity-weighted technique can be analyzed in the following two common cases in SAR-PU algorithm, namely, the propensity score is known and the propensity score is unknown.

When the propensity score is known, the propensity weighted estimator is calculated with the propensity score as:

(9) $$R\left(\bar{y}|y\right)=\frac{1}{n}\sum_{i=1}^{n}y_{i}\delta_{1}\left({\bar{y}}_{i}\right)+\left(1-y_{i}\right)\delta_{0}\left({\bar{y}}_{i}\right)$$

where $R(\bar{y}|y)$ is a propensity weighted estimator, y and $\bar{y}$ are actual label and observed label of instances respectively, and $\delta_{0}$ and $\delta_{1}$ are the costs for predicting an instance as negative and the positive accordingly.

It is worth mentioning that in most cases, the actual propensity score e(x) is unknown but the propensity score on the basis of observed labels $\hat{e}$ can be estimated. When the propensity score is unknown, the bias propensity-weighted estimator can be calculated as:

(10) $$bias\left(\hat{R}\left(\bar{y}|\hat{e},s\right)\right)=\frac{1}{n}\sum_{i=1}^{n}y_{i}(1-\frac{e_{i}}{{\hat{e}}_{i}})\delta_{1}\left({\bar{y}}_{i}\right)-\delta_{0}\left({\bar{y}}_{i}\right)$$

where n is the size of training set and $bias(\hat{R}\left(\bar{y}|\hat{e},s\right))$ is the biased propensity-weighted estimator.

In the presence of bias, the accuracy of the propensity score of only positive instances really matters. When the predicted class has extreme values (1 or 0), the incorrect propensity scores may have a greater impact. The incorrect value of propensity score can cause higher bias in the model.

### 4.2 Bayesian optimal relabeling

Bayesian Optimal Relabeling is the second well-known type of algorithms to achieve the instance-dependent PU learning. Probabilistic Gap Positive Unlabeled (PGPU) algorithm is the algorithm of this category, which is developed by He et al. [27]. They used SAR labeling mechanism in their proposed PGPU algorithm in which an instance to be labeled depends on its characteristics. The prime idea of this algorithm is that if an instance is more difficult to be labeled, then the probability of mislabeling of that instance will be larger. PGPU is based on an inadequate supposition that the positive instances nearer the latent optimal classifier are more difficult to be labeled. PGPU algorithm is based on the method for exploiting unlabeled instances of PU data introduced in Section 2.2, in which all unlabeled instances are treated as negative [56], [57]. The labels of these instances are consistent with the positive and negative instances allocated by Bayesian optimal classifier [58], [59].

The difficulty of an instance to be labeled can be estimated by the probabilistic gap ΔP(x). Following are four suppositions derived from Probabilistic Gap Positive Unlabeled (PGPU) algorithm:

(11) $$\left\{ \begin{array}{c} P(\bar{y}=-1|x,y=-1)=1\\ P(\bar{y}=+1|x,y=-1)=0\\ P\left(\bar{y}=-1|x,y=+1\right)=p_{1}\left(x\right)>0\\ P\left(\bar{y}=+1|x,y=+1\right)=1-p_{1}\left(x\right)>0 \end{array}\right.$$

where y and $\bar{y}$ are respectively the actual label and the observed label of an instance, and $p_{1}\left(x\right)$ is the mislabeled rate of positive instances.

Since the actual labels are not accessible directly due to the missing or noisy data, the actual probabilistic gap cannot be calculated directly. Therefore, they calculated the observed probabilistic gap first, and then correlate it with the actual probabilistic gap.

Probabilistic gap represents the distance of a positive instance from the decision boundary. If an instance is close to the decision boundary, it will be more difficult to be labeled. The Bayesian optimal classifier assigns a label to each instance with the maximum posterior probability [60]. It is a significant feature of probabilistic gap that it can be used as a Bayesian optimal classifier for PU datasets. The Bayesian optimal classifier can be expressed as following in binary classification conditions:

(12) $$\bar{y}\left(x\right)=\left\{ \begin{array}{c} +1,\;P_{+}-P_{-}>0\\ randomly\;selection,\;P_{+}-P_{-}=0\\ -1,\;P_{+}-P_{-}<0 \end{array}\right.$$

where $P_{+}=P\left(y=+1|x\right)$, $P_{-}=P\left(y=-1|x\right)$, and $P_{+}-P_{-}=0$ is the threshold for a classifier. According to PGPU algorithm, the mislabeled rate p(x,y) is a monotone decrease function regarding their respective probabilistic gaps.

They corrected the bias by using Kernel Mean Matching (KMM) technique [61] in their algorithm. In the end, the boundary can be estimated by following two methods: 1) Calculating the average of $\bar{n}$ smallest $\Delta \bar{P}\left(x\right)$, and 2) Finding the boundary through cross-validation.

## 5 Related fields of instance-dependent PU learning

Instance-dependent PU learning is closely related to some other typical fields of machine learning. In this section, we discuss the two most related areas of instance-dependent PU learning including instance-dependent label noise learning and cost-sensitive learning.

### 5.1 Instance-dependent label-noise learning

In many approaches of instance-dependent PU learning, unlabeled instances are considered as negative with label noise. As such, instance-dependent PU learning is transformed to an instance-dependent label noise learning problem [62], [63], [64]. In this sense, instance-dependent PU learning is a specific scenario of instance-dependent label noise learning with only false negative noise. That is to say, instance-dependent PU learning is a binary classification problem in which label noise only exists in one class, hence it is also known as one-sided instance-dependent label noise learning [65].

The algorithms of instance-dependent label noise learning commonly consider realistic noises in the label space [63], [66], where the probability of an instance being mistakenly labeled depends on both classes and its features. It is worth mentioning that such noise is quite common in real-world scenarios [67], [68]. In real-world situations, the poor-quality instances or the uncertain instances are more likely to be mislabeled [69], [70].

For example, the handwritten digits for training a recognition model are often manually annotated. It is apparent that legible handwritten digits are easier to label than the ambiguous ones. Noise is very likely to appear in ambiguous handwritten digits. The same assumption may also be observed in various practical applications, such as speech recognition, spam filters, pattern recognition, hyperspectral imaging, etc.

### 5.2 Cost-sensitive learning

Cost-sensitive learning is also closely related to instance-dependent PU Learning. Cost-sensitive PU learning is a well-known method for exploiting unlabeled instances in traditional PU learning as already discussed in Section 2.2. In cost-sensitive learning, instances are re-sampled and re-weighted according to the costs regarding different classes [71], [72]. In cost-sensitive learning technique, different weights are assigned to different training instances either manually or automatically. Here, we only focus on determining the cost of misclassified data. By reweighting the training instances, the erroneous data distribution observed in training set can be calibrated to a possible correct one, so that the ideal data distribution can be estimated [73], [74], [75]. Weighted logistic regression [26] and weighted SVM [17] are very common techniques employing cost-sensitive learning for PU learning. These techniques regulate the data weights by applying different regularization parameters to the positive-labeled instances and unlabeled instances. It is worth mentioning here that adjustment of regularization parameters is usually based on personal experience or heuristic rules which may lead to unsatisfactory performance. In order to solve the problems associated with the improper adjustment of the parameters, some recent works have focused on designing various unbiased risk estimators that can achieve improved performance. Specifically, Du Plessis et al. [20] proposed a non-convex ramp loss to rectify data bias due to the lack of negative instances and to overcome the defect of non-convexity. The key idea of unbiased convex loss is to use weighted compound convexity and weighted regular convex loss function to exploit unlabeled data.

## 6 Experiments

To compare the performance of the existing instance-dependent PU learning algorithms, in this section, we perform intensive experiments over the aforementioned well-known instance-dependent PU learning methods as well as traditional PU learning methods. To be specific, the instance-dependent PU learning algorithms incorporated for comparison include PUSB [24], SAR-PU [53], and PGPU [27], which have been introduced in Section 4. Moreover, three well-known traditional instance-independent PU learning methods are also employed for our comparison, which are:

• **WPU** [17]**:** Weighted Positive Unlabeled (WPU) is a well-known traditional PU learning algorithm, which argues that a classifier trained on PU examples predicts probabilities that differ by only a constant factor from the true conditional probabilities of being positive.

• **uPU** [20]**:** Unbiased Positive Unlabeled (uPU) is also a state-of-the-art traditional PU learning algorithm, where an unbiased risk estimator for PU learning is proposed.

• **nnPU** [21]**:** Non-negative Positive Unlabeled learning is also a well-known traditional PU learning algorithm, which improves uPU algorithm by eliminating the over-fitting problem induced by the negative empirical risk.

### 6.1 Experiment on synthetic dataset

**Fig. 1:**
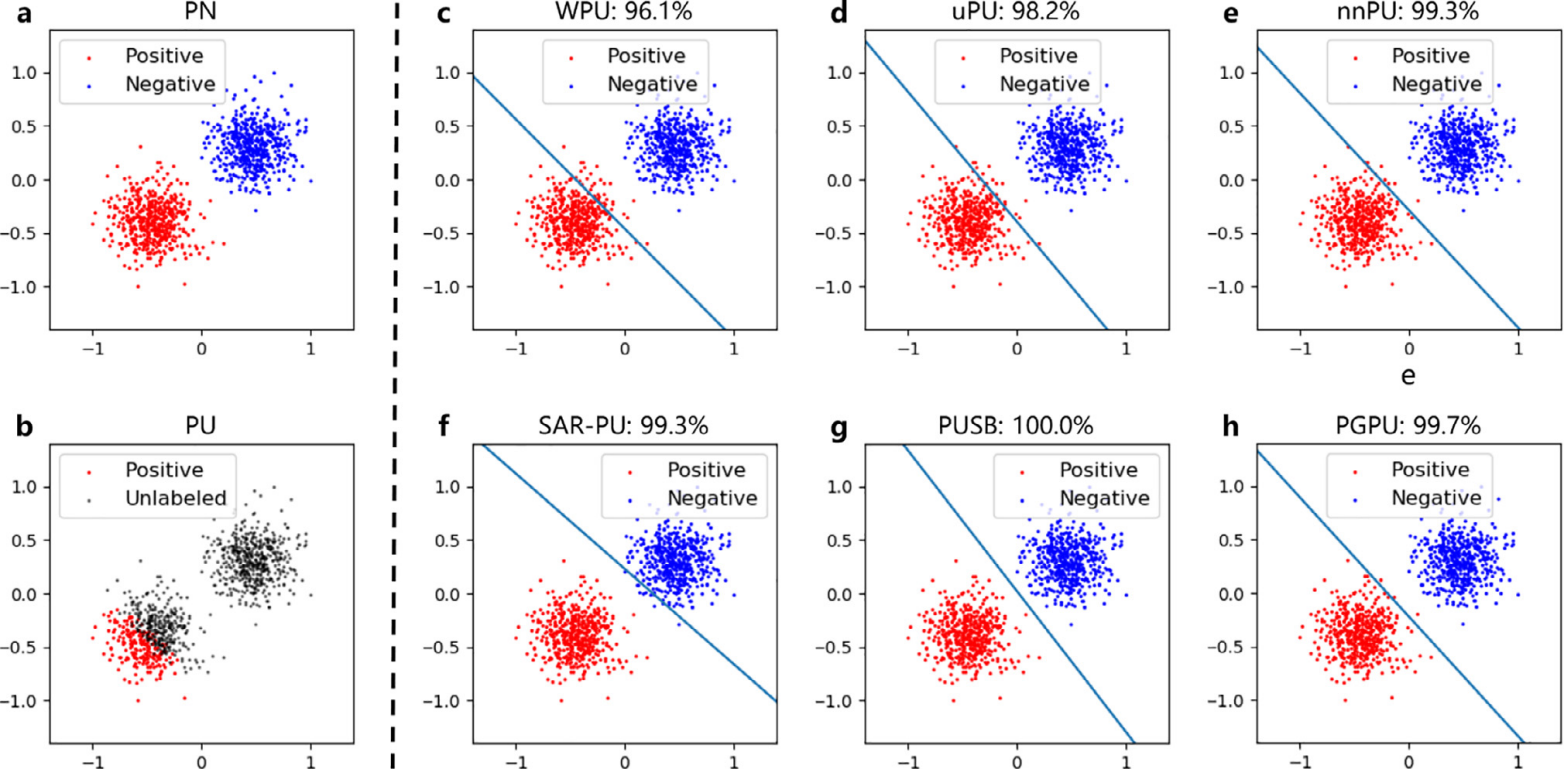
**The performances of various methods on the synthetic dataset.** (a) The real positive and negative data; (b) shows the generated positive and unlabeled data; (c-h) display the classification results generated by WPU, uPU, nnPU, SAR-PU, PUSB, and PGPU, respectively. The classification accuracy of every method is presented above the corresponding subfigure.

To visualize the performance of various PU methods, we adopt a synthetic 2-D dataset termed *TwoGaussian* appeared in [11] for our experiment. This dataset consists of two clusters of data generated from two Gaussians, and each Gaussian corresponds to a class (*i.e.*, positive/negative) as shown in Fig. 1a. The centers of two Gaussians are (1,0) and (−1,0), respectively, and their variances are set to 1. The entire dataset contains 1000 data points, which are equally divided into two classes. After that, a set of positive examples are sampled in a biased way by following the strategy in [11]. The proportion of selected positive examples, *i.e.*, c, is set to 0.4, and the selected positive data and unlabeled data are shown in Fig. 1b, which suggests that whether a positive example is labeled relies on its location, and the positive example that is far from the potential decision boundary is more likely to be labeled.

The classification results of all compared methods are shown in Fig. 1e–h. For the instance-independent algorithms such as WPU, uPU, and nnPU, a considerable number of data points are mis-classified due to the biased sampling of positive examples. For example, in Fig. 1c–e, some unlabeled examples that are originally positive near the decision boundary are classified as negative by WPU and uPU. By contrast, the instance-dependent methods usually achieve relatively better performance. For SAR-PU, the labels of all positive examples are correctly predicted, even though several negative data points near the decision boundary are mislabeled. PGPU and PUSB achieve nearly perfect performance, *i.e.*, 99.7% and 100% accuracy. Generally, the instance-dependent methods show better performance than the instance-independent ones, which suggests the superior ability of instance-dependent algorithms in dealing with the labeling bias on positive data.

### 6.2 Experiment on UCI benchmark dataset

In this section, we adopt five typical datasets from UCI machine learning repository [76], namely *Adult, Breast Cancer, Image Segmentation, Mushroom*, and *Splice*, to evaluate the performance of all investigated PU learning algorithms. The brief description of all UCI benchmark datasets used in our experiments is given in Table 4, which indicates the number of instances n, the feature dimensionality d, the number of positive instances $n_{+}$, the number of negative instances $n_{-}$, and positive class prior P(y=+1) in each dataset.

**Table 4:** **Characteristics of datasets from UCI machine learning repository**.

Dataset	n	d	$n_{+}$	$n_{-}$	P(y=+1)
*Adult*	48,842	14	23,520	25,322	48.2%
*Breast Cancer*	683	10	143	540	20.9%
*Image Segmentation*	2310	19	1024	1286	44.3%
*Mushroom*	8124	112	3916	4208	48.2%
*Splice*	3190	61	1478	1712	46.3%

For each dataset listed in Table 4, we make five subsets of whole dataset with almost equal size, which facilitates the subsequent five-fold cross validation. In each training round, we use 80% of the original instances for training and the remaining 20% are used for testing. In our experiments, we construct the instance-dependent PU datasets manually from the original UCI benchmark datasets. To be specific, we first train a Bayesian optimal classifier with the ground-truth labels of the training set, and then we can obtain the posterior probability P(y=+1|x) for each training instance. As aforementioned, the positive instances that are closer to the potential decision boundary (*e.g.*, smaller P(y=+1|x)) are less likely to be labeled. Based on this intuition, for each dataset, c={20%,30%,40%} of the positive training instances are selected to form the labeled positive set, where each positive training instance will be chosen with the corresponding probability P(y=+1|x). The remaining positive training instances and all negative instances are considered unlabeled. Under each c, the formation of the training set is kept identical to all compared methods to ensure fair comparison.

The experimental results on UCI benchmark datasets are presented in Table 5. It can be seen that on all datasets with different values of c, the instance-dependent PU learning methods (*i.e.*, PUSB, SAR-PU and PGPU) generally outperform the traditional instance-independent approaches (*i.e.*, WPU, nnPU and uPU), which demonstrate the advantage of the investigated instance-dependent PU learning methods over instance-independent approaches. The reason is that instance-dependent PU explicitly takes the labeling bias of positive data into consideration, which is beneficial for establishing an accurate classifier. Moreover, we see that PGPU usually achieves the top-level performance in most cases. This is because that PGPU relates the “difficulty” of labeling a positive example to its labeling probability, and the gap between Bayesian posteriors P(y=+1|x) and P(y=−1|x) is employed to model such difficulty.

**Table 5:** **Comparison of averaged classification accuracies** (%) **with a standard deviation of existing instance-dependent PU learning algorithms and the traditional PU learning algorithms on UCI benchmark datasets.** The best record under each c is marked in **bold**.

Datasets	c	**WPU**[17]	**nnPU**[21]	**uPU**[20]	**PUSB**[24]	**SAR-PU**[53]	**PGPU**[27]
*Adult*	20%	80.44 ± 2.2	80.22 ± 2.1	81.71 ± 2.2	82.57 ± 2.8	82.64 ± 2.6	**82.93**±**1.8**
	30%	80.13 ± 2.0	80.05 ± 2.0	82.41 ± 2.0	82.14 ± 1.9	**82.93**±**2.9**	82.76 ± 1.8
	40%	80.01 ± 1.9	80.74 ± 2.1	82.04 ± 2.1	**82.91**±**1.7**	82.53 ± 2.8	82.79 ± 1.9
*Breast Cancer*	20%	84.51 ± 2.5	84.80 ± 2.6	86.06 ± 2.6	86.38 ± 2.1	86.42 ± 2.7	**87.00**±**1.7**
	30%	84.23 ± 2.6	84.45 ± 2.4	85.85 ± 2.7	86.94 ± 2.0	86.75 ± 2.9	**87.13**±**2.0**
	40%	84.10 ± 1.4	84.33 ± 1.9	86.33 ± 1.8	**87.49**±**1.9**	87.12 ± 2.7	87.34 ± 2.1
*Image Segmentation*	20%	75.22 ± 1.8	76.05 ± 2.1	76.85 ± 2.0	78.53 ± 1.4	78.44 ± 2.3	**78.77**±**1.9**
	30%	71.80 ± 1.7	72.67 ± 2.0	73.51 ± 1.2	78.26 ± 2.3	78.32 ± 2.7	**78.51**±**1.6**
	40%	64.77 ± 1.9	66.41 ± 2.1	65.19 ± 2.2	78.49 ± 2.2	78.17 ± 1.9	**78.51**±**1.8**
*Mushroom*	20%	78.13 ± 2.0	79.01 ± 1.2	79.67 ± 2.1	80.07 ± 1.8	**80.42**±**1.0**	80.39 ± 2.5
	30%	78.98 ± 2.1	78.96 ± 1.0	79.32 ± 2.3	80.17 ± 1.7	80.69 ± 1.9	**81.03**±**2.5**
	40%	78.13 ± 2.0	78.91 ± 1.2	79.14 ± 3.0	80.69 ± 2.5	80.76 ± 1.5	**80.89**±**2.5**
*Splice*	20%	56.72 ± 1.8	57.12 ± 1.1	57.12 ± 2.1	58.18 ± 1.4	**58.90**±**1.9**	57.32 ± 2.1
	30%	56.72 ± 1.5	56.83 ± 3.0	57.71 ± 3.0	**58.63**±**1.6**	58.31 ± 1.8	58.11 ± 2.0
	40%	55.74 ± 1.7	56.03 ± 3.1	56.33 ± 3.0	**59.09**±**1.5**	58.48 ± 2.1	58.53 ± 1.9

### 6.3 Experiment on real-world dataset

We further investigate the performance of typical PU methods including WPU, nnPU, uPU, PUSB, SAR-PU, and PGPU in tackling real-world applications. To this end, we use *CIFAR-10* dataset and extract the images of “cat” and “dog” for our experiment [5], and the target is to classify every test image example into one of the above two classes. Similar to the experiments in Section 6.2, we also generate the positive examples according to the posterior P(y=+1|x) output by a Bayesian optimal classifier. To be specific, we first train a Multi-Layer Perceptron (MLP) on training data with original real labels. Then, we pick up the positive data according to the predicted probabilities given by MLP. Besides, five-fold cross validation is conducted on all compared methods, and their mean test accuracies and standard deviations are recorded to investigate the ability of the compared methods in image classification.

For all compared baseline methods, we take ResNet-18 [77] as the backbone network. The parameters of every algorithm have been carefully tuned to achieve the best performance. For uPU, we choose the regularization parameter λ from $\left\{10^{-3},10^{-2},\cdots ,10^{1}\right\}$. In nnPU, the step discounted parameter γ and the tolerance parameter β are respectively set to 0.001 and 0 as suggested by [21]. In PUSB, the density ratio r(x) is estimated via minimizing the pseudo classification risk. In PGPU, the boundary ℓ is estimated by calculating the mean of $n^{\prime }$ smallest probabilistic gap and the value of $\beta \left(x\right)=\frac{P_{D}\left(x\right)}{P_{D*}\left(x\right)}$ is obtained by the kernel mean matching (KMM) technique. Note that uPU and nnPU require the positive class prior P(y=+1), and here we simply assume it to be known and feed the real positive class prior to these algorithms during training.

From the experimental results reported in Table 6, we see that PUSB achieves the best performance among all methods under all labeling cases. In general, instance-dependent methods (*i.e.*, PUSB, SAR-PU and PGPU) outperform the instance-independent ones (*i.e.*, WPU, uPU, and nnPU) in most cases, which again shows the necessity of instance-dependent PU learning algorithms.

**Table 6:** **Comparison of averaged classification accuracies** (%) **of existing instance-dependent PU learning algorithms and the traditional PU learning algorithms on real-world*****CIFAR-10*****dataset.** The best record under each c is marked in **bold**.

Dataset	c	**WPU**[17]	**uPU**[20]	**nnPU**[21]	**PUSB**[24]	**SAR-PU**[53]	**PGPU**[27]
*CIFAR-10*	20%	91.00±0.98	92.22±0.34	91.82±0.21	94.35±0.56	92.73±0.18	93.42±0.53
30%	93.41±0.39	94.38±0.26	93.24±0.18	95.42±0.81	94.28±0.15	95.21±0.46	
40%	93.31±0.81	95.21±0.45	95.32±0.32	96.31±0.72	95.62±0.42	95.67±0.39	

## 7 Conclusion

In this survey, we comprehensively review the recent research advances in instance-dependent PU learning. Starting from the introduction of general PU learning, we then detail some important aspects regarding instance-dependent PU learning, which include the commonly adopted labeling assumptions (*i.e.*, “selected completely at random” and “selected at random”), two main types of algorithms (*i.e.*, “scoring function algorithms” and “Bayesian optimal relabeling”), and the strongly related fields (*i.e.*, instance-dependent label-noise learning and cost-sensitive learning). Finally, we provide some empirical comparisons of representative PU learning methods on some synthetic, UCI benchmark and real-world datasets, which suggest that instance-dependent PU learning usually have better performance than traditional instance-independent PU learning in dealing with practical data.

Due to the huge practical demand, we believe that instance-dependent PU learning will gain more attention from both academic and industrial circles. We believe that the following directions are worth further studying:

(1) Existing methods usually rely on some pre-defined labeling assumptions (*e.g.*, SCAR or SAR). However, for a real-world application, we do not know the actual labeling process in advance. Therefore, we need to design new algorithms that are free from assumptions, or are automatically adaptive to different assumptions.

(2) How to accurately and explicitly model the relationship among s, x and y is still a challenging yet important problem. Although some formulations, such as propensity score, have been defined, the exploration on their relationship is still inadequate.

(3) As an important branch of weakly-supervised learning [31], instance-dependent PU learning is quite general and can be applied to various domains such as computer vision, geoscience, financial data analysis, and medical science. Therefore, the applications of instance-dependent PU learning to different kinds of practical problems are also worth investigation.

## Declaration of competing interest

The authors declare that they have no conflicts of interest in this work.
